# Substance Misuse Among Medical Students, Resident Physicians, and Fellow Physicians: A Review With Focus on the United States' Population

**DOI:** 10.7759/cureus.72636

**Published:** 2024-10-29

**Authors:** Troy Salisbury, Christopher Chamanadjian, Han Nguyen

**Affiliations:** 1 Psychiatry, Loma Linda University Medical Center, Loma Linda, USA; 2 Psychiatry, Charles R. Drew University of Medicine and Science, Los Angeles, USA; 3 Psychiatry, Los Angeles General Medical Center and University of Southern California, Los Angeles, USA

**Keywords:** fellow physicians, graduate medical education (gme), medical students, resident physicians, spiritual health, substance addiction, substance misuse, substance treatment utilization, substance use disorder, substance use policy

## Abstract

Substance misuse among medical trainees is a serious ongoing concern. The goal of this review is to describe the contributing factors leading to substance misuse among medical trainees, the consequences for those with substance misuse issues, and explore potential interventions and policy changes to address the issue. There are demographic characteristics that contribute to a higher likelihood of substance misuse including race, sex, relationship status, and year of training. Loss of training position, legal consequences, medical errors, and premature death are some of the negative effects more likely for those with substance use issues. There is a lack of clarity regarding institutional substance use policies and implementation of standardized, evidence-based policies is limited. There are many barriers preventing those with substance use issues from seeking care. Educational sessions targeted at recognizing signs of substance misuse and identification of resources have shown efficacy, while spiritual health support may be an effective area of intervention. There are many factors perpetuating substance misuse among this population. Strategies to address this issue should target improving the utilization of resources, updating institutional policies, and supporting spiritual health.

## Introduction and background

Substance use among medical trainees and physicians has been an area of ongoing concern and research as well as represents an international dilemma [1-4]. Medical students, residents, fellows, and attending physicians represent a group that is particularly important to study due to their access to controlled substances and the danger caused by practicing while impaired. This issue is especially of concern for medical trainees, who face intense work demands and often with little support [5].

Much of the discussion and landmark research related to substance use among medical students and physicians began in the 1990s, and it was found that despite lower rates of other substance use compared to the general population (including lower use rates of tobacco, marijuana, and cocaine for medical students; marijuana, cigarettes, cocaine, and amphetamines for female residents; marijuana, cigarettes; cocaine, amphetamines, psychedelics, LSD, barbiturates, and heroin for male residents), surveyed medical students, residents, and attending physicians reported equal or higher rates of use of alcohol and benzodiazepines [6-8]. Importantly, substance misuse, the use of substances in ways that produces harm to a person or those around them, has been shown to worsen when beginning medical school and residency, a finding still demonstrated by recent literature published in 2020 [6,9].

More recent research has demonstrated that substance misuse continues to be an ongoing area of concern among this population [1,10-13]. Although misuse of prescription drugs and use of illicit drugs is now rare among physicians (1.3%), alcohol misuse is widespread with a prevalence of one-third of medical students, half of residents, and 12.9% of attending male physicians and 21.4% of female physicians [1,10-13]. The purpose of this review is to provide an evidence-based update regarding the contributing factors leading to substance misuse in this population, the pervasiveness of this issue and the severity of consequences for those with substance misuse, and potential ways of addressing this complicated problem.

## Review

Contributing factors

Medical Students

There is a discrepancy in alcohol misuse among residents and medical student demographic lines [11,12]. Sex and race are significant predictors of substance misuse among medical students. Surveyed male medical students engaged in alcohol misuse in the form of binge drinking and other substance use more often than their female peers [12]. Additionally, Caucasian students consumed alcohol more frequently than non-Caucasian students. Age and relationship status have also been identified as associated factors with substance misuse. Younger students and those who are single were more likely to misuse alcohol [13]. Financial burden appears to be a contributing factor as well because students with larger educational debt were more likely to misuse alcohol. Importantly, similar characteristics among residents as demonstrated among medical have been associated with substance misuse [11,14].

Resident Physicians

As with medical students, many contributing factors have been found to be associated with substance misuse among surveyed resident physicians (Table 1) [11,14]. Sex and race are significant predictors of substance misuse among residents. One self-reported survey demonstrated male resident physicians are 2.5 times more likely to misuse alcohol [11]. Another study recently published in 2020 demonstrated that male residents were 100 times more likely to misuse alcohol compared to their female colleagues [14]. Also, those of Asian race in comparison to white race were found to be 31% as likely to misuse alcohol, and this was the only statistically significant difference identified when races were compared (white vs non-white, black vs white, and other than Asian or black vs white did not show significant differences). Likewise, residents with a relationship status of single or divorced compared to married demonstrate 2.3 times higher rates of substance misuse [14]. The amount of time spent in practice is also an influencing factor. Residents who are more senior in their residency training are 25% as likely to misuse substances compared to their junior counterparts, although age itself was not associated with changes in rates of substance misuse [11]. Alcohol misuse is a common struggle across the spectrum of practice from medical students to attending physicians, but there is one group of substances that are uniquely utilized to a significant extent by medical students and not by physicians [15].

**Table 1:** Comparative likelihood of substance misuse among resident physicians across demographic variables that have statistical significance [11,14]

Demographic variable	Comparative likelihood of substance misuse (odds ratio)
White vs non-white	0.55
Asian vs white	0.31*
Black vs white	0.86
Other than Asian or black vs white	0.62
Male vs female sex	2.5*-100*
Single or divorced vs married relationship status	2.3*
Senior vs junior level in residency training	0.25*
Age (each additional year)	0.88

Cognitive Enhancers

Psychostimulants are perceived to improve cognitive performance and the use of psychostimulants such as methylphenidate as cognitive enhancers indicates a distinctive difference in substance use patterns among medical students and physicians [1,15]. Survey data demonstrates that the use of psychostimulants has been increasing among medical students [15]. In this USA-based study, almost 20% of surveyed medical students have used prescription psychostimulants at least once, with first use most often in their undergraduate studies. Furthermore, over 10% of students reported use during medical school. Misuse of these medications was common such that 63% of students had used psychostimulants not prescribed to them and 23% of those who had received a prescription for psychostimulants had diverted the psychostimulant prescribed to them. Competitive pressure may play a role in motivation for use. Students training at a medical school that determined class rank were more likely to misuse cognitive enhancers among those surveyed in USA medical schools. It is possible that a medical student’s desire to be a more competitive residency applicant to match their desired specialty could be a contributing factor, as medical specialty choice is associated with substance use patterns [1,16].

Medical Specialty

Physicians’ chosen medical specialty has been linked to substance misuse patterns [1,16]. Some more dated research demonstrated that compared to the national average of all residents surveyed, emergency medicine residents reported higher rates of cocaine use in the past year (prevalence=14%, p<0.05) and higher use rates of marijuana (prevalence=28.6%, p=0.03), and psychiatry residents reported the highest use rates of benzodiazepines (prevalence=27.3%, p=0.0001) and marijuana (prevalence=34.1%, p=0.0001)[16]. Of note, rates of substance use were similar but not identical to the rates among surveyed attending physicians in these specialties published at a similar time [17]. However, more recent substance use data from among attending physicians has shown differences compared to the findings just discussed [1].

The discrepancy in substance misuse rates along specialty choice for surveyed attending physicians still exists today, but the predominant substance of misuse currently for attending physicians across all specialties is alcohol [1]. In the absence of more recent substance use data from resident physicians based on specialty choice, this data showing a change in attending substance use patterns over time may indicate that a similar change has also occurred among resident physicians. Further elucidation of the contributing factors to substance use is of great importance, as the implications for those with substance misuse are serious [18].

Implications

Training Program-Specific Repercussions

The policies for dealing with a positive drug test of their medical trainees vary among each residency program [18]. Among surveyed programs, program directors report it is much more common that a medical student would be denied their position than be allowed to continue with special conditions. In contrast, an almost equal number of programs would allow a resident to continue training with special conditions as would revoke a resident’s seat in the program. We advocate for more consistent and non-punitive policies but is important to note that repercussions resulting from a medical trainee’s program identifying their substance misuse is only one part of the consequences that occur as a sequela of substance misuse [12,13,18-20].

General Consequences

There are serious consequences for medical trainees with substance misuse for their personal health, academic and professional success, as well as legally [12,13,18-20]. Medical students reported hangovers, regretting choices, engaging in fights, missing class, performing poorly on important projects or tests, driving a car under the influence, and other consequences all due to substance use [12]. Furthermore, alcohol misuse among medical students is associated with burnout, emotional exhaustion, depersonalization, and depression [13]. In addition to the previously stated risks, the risks for those with substance misuse also include the most serious and irreversible outcomes [19].

Based on a retrospective cohort study of resident and fellow data reported to the American Board of Anesthesiology by program directors, those with substance misuse have a greatly increased risk of death during or after completion of their training, adverse training outcomes (including failure to complete their training or become board certified), and adverse medical licensure actions after training [19,20]. Warner et al. identified that more than 7% of the residents and fellows they studied with substance misuse died during training compared to zero of those without [19]. At the end of follow-up, more than 14% of those with substance misuse were deceased compared with 1.3% of those without. The negative outcomes associated with substance misuse among medical trainees continue beyond years of training, as they are similar to those studied in attending physicians as well.

Sequela for Attendings

Attending physicians with alcohol misuse suffer serious consequences throughout various areas of their lives [1]. Alcohol misuse among attendings was associated with burnout, depression, suicidal ideation, lower quality of life, lower career satisfaction, and recent medical errors. A study of anesthesiology attendings published in 2020 found that among, those with substance misuse, nearly 20% were deceased from a substance misuse-related cause [20]. The consequences of substance misuse for medical trainees and attendings are grave and to help minimize the fallout in providers’ lives and their patient care, this issue must be further addressed.

Addressing the issue

Access Barriers

There are many barriers that limit a medical trainee’s willingness to seek care when they are facing mental health problems [4,21-23]. These barriers are of great significance because, among medical trainees, the prevalence of mental health problems, including substance misuse, that needed treatment over the preceding year increased throughout years of medical training starting from the first year of residency to the end of residency training [4]. However, there was a trend of decreased help-seeking over time in the same study as well as in another recent study of residents and fellows that conducted a retrospective analysis of medical records from 2021 [4,24].

An additional recent study published in 2021 identified that surveyed younger doctors (those who are 40 years old or younger, including residents) are less likely to seek support or treatment for problems related to their mental health [23]. Furthermore, younger practitioners are most likely to report barriers to seeking mental health treatment including confidentiality and impact on career progression. This last finding may explain why there is decreased help-seeking overtime among medical trainees, yet older doctors are more likely to seek help. As medical trainees advance through residency and approach independent practice, the potential impact on career progression would be a greater barrier to help-seeking in the early, fledging stages of their career rather than when they are older, later in their career, and more likely to be established in their desired positions. Overall, these studies reveal that despite the need for care throughout training, medical trainees often do not seek help, demonstrating a need to break down the barriers faced by these individuals in utilizing resources.

Many obstacles to seeking mental health resources have been identified specifically among resident physicians and include both individual and structural barriers and have been reported in research as recent as 2021 [21,22]. Individual-level barriers, including the stigma associated with having a mental illness, are commonly cited by surveyed residents as contributing to their reluctance to seek help. This includes stigma that may be experienced personally or from concerns regarding negative career consequences for physicians with mental health problems. Additionally, personality traits appear to play a role in the likelihood of a medical trainee to seek help for mental health problems [4]. Reality weakness personality traits include characteristics such as paranoid traits, identity security, and problems with relationships. Predictors of help-seeking among medical trainees are higher perceived levels of mental health problems and a low level of reality weakness personality trait.

Structural barriers to help-seeking are also present, including lack of formal support for mental health issues by way of access to or promotion of resources, inadequate time to seek help, and prohibitory cost of treatment [21,22]. These barriers might explain the resident preference for seeking support informally from peers, friends, and family [22]. However, among residents who do seek help, utilization of outpatient mental health resources has been shown to be highest for females, first-year residents, and general internal medicine residents [24]. Resident physicians under-utilize mental healthcare resources, a finding that has been demonstrated among surveyed attending physicians as well, signifying the continuation of mental health issues identified among residents beyond residency years [25]. Tyssen et al. found that two-thirds of attending physicians with mental health disorders were self-treated, including self-prescription of tranquilizers. This is particularly concerning, considering reports of physician substance misuse beginning with benzodiazepines, and then graduating to more dangerous controlled substances [26]. One of the barriers to attending physicians seeking help may be fear of the negative impact on their credentialing if it was discovered they have a problem with substance misuse given that, as previously described, there is an increased rate of adverse medical licensure actions for physicians with substance misuse after residency. Many obstacles exist that decrease the likelihood that medical trainees will obtain the mental health resources they need, underscoring the importance of institutional interventions to address this issue.

Interventions

For those with substance misuse in the medical education pathway, it is important to get help as soon as possible [27]. There is a scarcity of literature concerning the treatment of substance use among medical trainees. However, some research exists outside of a specific medical training context that can serve as a model for how institutional interventions can be effective in treating medical trainee substance misuse. Stockings et al. demonstrated that early intervention of substance misuse among young adults in the setting of an individual’s university or workplace has shown to be effective in the reduction of negative outcomes, particularly those related to alcohol misuse. It is therefore plausible that clear, consistent institutional interventions and policies regarding substance misuse would benefit medical trainees.

Efforts are being taken by different institutions to improve the issue of substance misuse among residents [28]. One program created an educational resource reviewing burnout, substance misuse, and mood disorders for their residents. This resource included an online module and a role-play scenario. In their 2021 publication, they demonstrated that the surveyed residents who participated in the role-play activity outperformed their peers who did not participate when identifying signs of mental health issues, including substance misuse. They also reported increased confidence in identifying resources for themselves and their peers. With promising results exhibited in this limited sample, the implementation of an online module and role-play scenario could be considered for more residency programs to bridge the gap between barrier access and limited education. Other avenues for intervention could also be beneficial, such as measures to promote spiritual health [9].

Institutional interventions to support medical trainees’ spiritual health may be an effective strategy to reduce substance misuse among medical trainees on the premise that higher trainee spirituality is associated with lower substance use and misuse [9]. Importantly, though, there is a lack of literature evaluating the efficacy of medical institutions addressing substance misuse issues by targeting the spiritual health of medical trainees. Despite the insufficient literature assessing this strategy inside the context of medical trainees, research outside of this specific context has demonstrated efficacy for spiritual and religious interventions in helping people with substance use problems [29]. Institutional support for the spiritual well-being of medical trainees has the potential to be a promising area for future research as well as a new avenue for the prevention and treatment of substance misuse.

Policies

Many medical trainees are unclear about their institution’s existing policies and resources regarding substance misuse [12]. Three-quarters of surveyed medical students reported either their medical college did not have a substance prevention program or they were unsure if they did. Only a little more than half of medical students believe their medical college is concerned about the prevention of substance use. This uncertainty regarding substance policy also extends to institutional leaders. A majority of residency program directors at programs that drug test incoming residents do not know what actions would be taken in response to positive drug tests from incoming medical trainees [18]. Furthermore, the lack of awareness regarding institutional policies related to substance misuse by trainees and leadership underscores the absence of such policies as a whole [30].

Program directors’ uncertainty regarding their institutions’ substance use policy reflects that such policies among US medical schools are lacking in general, according to policy data published in 2021 obtained from more than 70% of US allopathic medical schools’ student handbooks [30]. Mannes et al. determined that half of the US allopathic medical schools met zero out of the six Association of American Medical Colleges guidelines for the development of chemical impairment policies. Only 1% of surveyed schools met all six guidelines.

The previously described marked inconsistency of program policy in response to positive drug tests and the limited awareness of policies that are in place combine to limit help-seeking among trainees because, as previously discussed, potential punitive responses by programs are a major reported barrier to help-seeking. We recommend that trainees with substance misuse be encouraged to seek help from their institutions and that the barriers cited by trainees be addressed. Specifically, we support consistent drug testing and other measures to help identify trainees with substance misuse since, as already described, early identification and intervention are important to recovery. However, punitive policies (e.g. dismissal from the program) toward those who are discovered to have substance misuse should be replaced with those that protect a trainee's position if the trainee is willing to engage in treatment and provide a pathway for returning to training after recovery. Additionally, policies should be regularly communicated to trainees and administrators as well as implemented consistently across different programs to maximize awareness. We suggest the implementation of recommendations based on the above findings in the key domains of institutional interventions, policy changes, and future research to address the issue of substance misuse among medical trainees (Table 2).

**Table 2:** Summary of recommendations for addressing substance misuse among medical trainees AAMC: Association of American Medical Colleges References Interventions: 1 [11-17], 2 [1,12,13,18-20,27], 3 [28], 4 [15]; Policies: 1 [12,18,30], 2 [18,27], 3 [4,18,21-24,27], 4 [4,12,18,21-24,27,30]; Future research: 1 [9,11-24,28,30], 2 [1,16,17], 3 [4,21-25], 4 [9,29]

Domain of recommendations	Specific recommendations related to the corresponding domain
Interventions	1. Medical trainees of the demographics shown to be at higher risk for substance misuse should be priority targets of institutional interventions.
	2. Interventions should seek to engage medical trainees as early as possible in their journey.
	3. The combination of online modules and role-play scenarios has demonstrated efficacy and should be implemented in more institutions.
	4. Psychostimulant misuse is an increasingly serious issue and warrants specific institutional resource allocation to mitigate.
Policies	1. Institutional policies should be regularly reviewed and communicated to medical trainees and administrators.
	2. Drug testing and other measures to identify trainees with substance misuse should routinely be done.
	3. Punitive policies toward trainees discovered to have substance misuse should be replaced with those that protect their position if treatment is accepted and provide a pathway to returning to training after recovery.
	4. Substance use policies should be consistent across institutions, incorporating national guidelines (such as AAMC’s) and other evidence-based practices (including those listed above).
Future research	1. Focus should be placed on collecting high-quality data utilizing more reliable data collection methods beyond that of self-report surveys.
	2. Updated research into the relationship between substance misuse and specialty choice is indicated to better characterize medical trainees most at risk for substance misuse and then to guide future interventions.
	3. Investigation should be conducted regarding how frequently reported barriers to help-seeking can be overcome to improve resource utilization by medical trainees.
	4. Interventions targeting spiritual and religious health to improve medical trainee substance misuse should be researched given the efficacy of such interventions in other contexts.

## Conclusions

Substance misuse among medical trainees continues to be an ongoing concern with limited data to date. This is particularly exhibited among certain demographic groups with discrepancies along lines such as age, race, sex, and spiritual practice. There are many harmful consequences for those with substance misuse in and outside of their medical training and career. Despite efforts to alleviate the continued high rates of substance misuse among medical trainees, there are still many factors preventing them from receiving help, as well as a paucity of literature related to it. More institutional interventions, policy changes, and research are needed to address this complicated problem.
